# Supplementary material for: Absence of a pressure gap and atomistic mechanism of the oxidation of pure Co nanoparticles
Source: Nat Commun. 2023 Jan 12;14:174. doi: 10.1038/s41467-023-35846-0 (PMC9837083; doi:10.1038/s41467-023-35846-0)
Supplement: Supplementary file 1 — Supplementary Information [file 41467_2023_35846_MOESM1_ESM.pdf]

Supplementary Information: Absence of a pressure gap and atomistic mechanism of the oxidation of pure Co nanoparticles

Jaianth Vijayakumar,[†] Tatiana M. Savchenko,[†] David M. Bracher,[†] Gunnar Lumbeeck,[‡] Armand Béché,[‡] Jo Verbeeck,[‡] Štefan Vajda,[¶] Frithjof Nolting,[†] C.A.F. Vaz,[*,†] and Armin Kleibert[*,†]

†*Swiss Light Source, Paul Scherrer Institut, 5232 Villigen PSI, Switzerland*

‡*EMAT, University of Antwerp, 2020 Antwerpen, Belgium*

¶*Department of Nanocatalysis, J. Heyrovský Institute of Physical Chemistry v.v.i., Czech Academy of Sciences, Dolejškova 2155/3, 18223 Prague, Czech Republic*

E-mail: carlos.vaz@psi.ch; armin.kleibert@psi.ch

Supplementary Discussion

1. Role of substrate on the oxidation of the Co nanoparticles

We approximate the morphology of the oxidized nanoparticle in state B by assuming a spherical, metallic Co core with diameter $D_c = 11\,\mathrm{nm}$ surrounded by a closed CoO shell with thickness $t_{\mathrm{oxi}} = 4\,\mathrm{nm}$, similar to the nanoparticles shown in the middle column in Fig. 3 of the main article. Using the atomic density of CoO, $n(\mathrm{Co}) = n(\mathrm{O}) = 50.7$ atoms per nm^3 (mass density $\rho = 6.31\,\mathrm{g} \cdot \mathrm{cm}^{-3}$) we obtain a value of 1.5×10^5 for the number of Co and oxygen ions present in the oxide shell. From the total number of Co atoms present in the core and oxide shell, we estimate a diameter of $D = 16.4\,\mathrm{nm}$ for the pristine spherical metallic Co nanoparticle. Such a nanoparticle provides a surface area $A = 841\,\mathrm{nm}^2$ for the adsorption of molecular oxygen. Based on the distribution of oxygen ions in the (001)-plane of the CoO lattice, we assume that a density of 13 sites per nm^2 is available for the adsorption of oxygen molecules and subsequent dissociation. With this assumption, the surface is saturated with 2.25×10^4 oxygen ions available to form a CoO-type mono-layer upon chemical reaction. Assuming a sticking coefficient of 1, such mono-layer is formed upon exposure of 1 L molecular oxygen. When further assuming that the oxide shell is formed by successive adsorption of oxygen and conversion into CoO monolayers, an exposure of at least 6 L is required to build up the $t_{\mathrm{oxi}} = 4\,\mathrm{nm}$ oxide shell. This exposure value is much larger than what we find experimentally (1–2 L for the nanoparticles deposited on SiO_x). In particular, since the actual sticking coefficient is likely to be smaller than 1 and is expected to be even smaller for the oxidized Co surface, such estimate for the required oxygen exposure should be seen as a lower limit. These considerations suggest that other sources of oxygen may be active, including from the substrate, where oxygen will also impinge and where atomic surface mobility combined with a sufficiently large diffusion length may provide an efficient source of molecular oxygen, as discussed in the main text. We can estimate the oxygen diffusion length, $\delta = 2\sqrt{D_s \tau}$, using values reported in the literature, where

$D_s = D_0 \exp(-E_{diff}/RT)$ is the diffusion constant, with E_{diff} the energy barrier for surface diffusion, T the temperature, and $R = 8.314\,JK^{-1}mol^{-1}$; $\tau = \tau_0 \exp(E_{des}/RT)$ is the average time an oxygen molecule stays on the surface before desorption, with $\tau_0 \sim 10^{-13}\,s$, and E_{des} is the desorption energy.[5] From temperature programmed desorption (TPD) spectroscopy the desorption energy can be estimated as $E_{des} \approx RT_{des}[\ln(T_{des}/\beta\tau_0) - 3.64]$, where β is the heating temperature rate and T_{des} the temperature of maximum oxygen desorption in the TPD spectrum;[6] for oxygen on silica, T_{des} has been measured to be 440 K at $\beta = 5\,Ks^{-1}$,[7] from which we obtain $E_{des} = 113\,kJmol^{-1}$ and $\tau \sim 4 \times 10^6\,s$. Values for the oxygen surface diffusion on silica have been determined for porous glasses[8,9] and for the surface diffusion of atomic oxygen in SiO_2.[10] The former studies likely refer to adsorbed molecular oxygen, but the adsorption energies corresponding to these processes, $15\,kJmol^{-1}$ or lower, suggest that they correspond to adsorption processes not relevant here; the latter study refers to atomic oxygen and is expected to become significant only at higher temperatures and not applicable to our case (in both instances, the estimated diffusion length are on the nm scale). To estimate the surface diffusion length for O_2 on SiO_2 we use the empirical correlation between the surface diffusion length and the desorption energy reported by Sladek *et al.*,[11] $D_s = 1.6 \times 10^{-6} \exp(-0.45E_{des}/mRT)\,m^2s^{-1}$, where $m = 1$ for non-polar molecules on insulating or conducting surfaces. Using this expression, one obtains $D_s = 2.4 \times 10^{-15}\,m^2s^{-1}$ at room temperature and $\delta = 200\,\mu m$. We can compare this value with that for the surface diffusion of molecular oxygen on Pt, $D_s = 2.5 \times 10^{-14}\,m^2s^{-1}$ at room temperature,[12] which yields $\delta \approx 700\,\mu m$. δ defines a radius around which oxygen can diffuse and be absorbed by the nanoparticle, defining an effective area which is orders of magnitude larger than the area exposed by the nanoparticle. The nanoparticle will act locally as a sink of surface oxygen, producing a concentration gradient that will act as a driving force for oxygen atoms further away from the particle. For the more inert carbon surface, various adsorption energies for oxygen have been reported that are found to depend strongly on the particular adsorption site.[13–18] For the temperature range most relevant to our experimental conditions, Bansal *et*

al.[13] have reported activation energies for oxygen chemisorption on graphitised carbon black to be in the range from 13–50 $\mathrm{kJmol^{-1}}$; from these values, we estimate a surface diffusion coefficient in the range from 1.65×10^{-7}–$1.93 \times 10^{-10}\,\mathrm{m^2s^{-1}}$ and surface residency times of 16 ps–50 μs, which give diffusion constants of 3 nm and 200 nm, respectively, much smaller than those found for SiO_x.

2. Size-dependent oxidation behavior in Co nanoparticles

Based on the proposed oxidation process, we can predict the oxidation behavior for both smaller and larger particles and compare with the available literature, at least as far as the final oxidation state is concerned.

For nanoparticles larger than those shown in the main text, we do not expect a different behavior. Indeed, larger particles in our study exhibit a very similar oxide shell evolution when compared to the smallest ones, as demonstrated in Fig. S5, which compares representative HAADF-STEM images for nanoparticles in the range from 9 to 27 nm in states B and C.

For smaller nanoparticles, we anticipate a size-dependent behaviour as follows:

(i) As discussed in the main text, the first oxidation step (CoO formation) from state A to state B is characterized by independent nucleation and growth of CoO crystals driven by very efficient surface diffusion of Co and oxygen until merging of the CoO crystals closes the shell. Also as discussed above in Section 1, we estimated that a core-shell particle with a metallic core with a diameter of 11 nm surrounded by a 4 nm CoO shell requires an initial spherical metallic nanoparticle with a diameter of 16.4 nm. This consideration suggests the first oxidation step consumes a metallic layer thickness of (16.4 nm - 11 nm)/2 $\approx$ 3 nm. Assuming that the nucleation density for CoO crystallite growth and the required metal thickness does not depend sensitively on particle size, we can conclude that metallic Co nanoparticles with diameters up to $\approx$6 nm are fully oxidized in the first oxidation step. This value corresponds well to the case where the particle size (radius) and the oxide crystallite

size are in a similar range and marks the lower particle size boundary for the onset of the Kirkendall effect.

(ii) The second, slower oxidation step towards state C is due to the Kirkendall effect, i.e., due to radial diffusion of oxygen and cobalt ions. To estimate how much Co is transferred from the metallic core to the oxide shell, we assume that the outer diameter of the void shell corresponds approximately to the original size of the metallic core in state B. We emphasize that we do not have direct access with TEM to the very same particle in states B and C. Our data (Fig. 3 of the main text) indicate that the thickness of the void shell is approximately 1 nm in width. Hence, we conclude that for nanoparticles with initial sizes between 6 and 8 nm the entire metallic core would be incorporated into the oxide shell after both oxidation steps to form a hollow oxide sphere, while for larger particles a yolk-shell structure results.

The predicted critical diameters discussed above, 6 nm for fully oxidized nanoparticles, 6–8 nm nm for hollow oxide spheres, and yolk-shell or core-shell structures for nanoparticles larger than 8 nm, agree in fact well with findings in the literature for nanoparticles oxidized under different conditions.[19–21]

Supplementary Figures

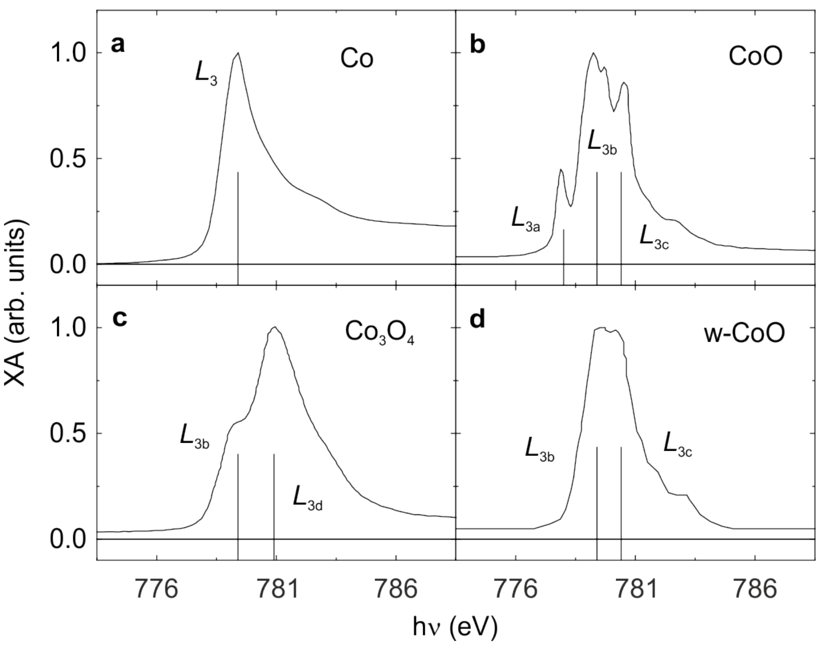

Figure S1: Reference X-ray absorption spectra used for the linear combination fitting analysis in this work for (a) Co,[1] (b) CoO,[2] (c) Co_3O_4,[3] and (d) wurtzite-CoO.[4]

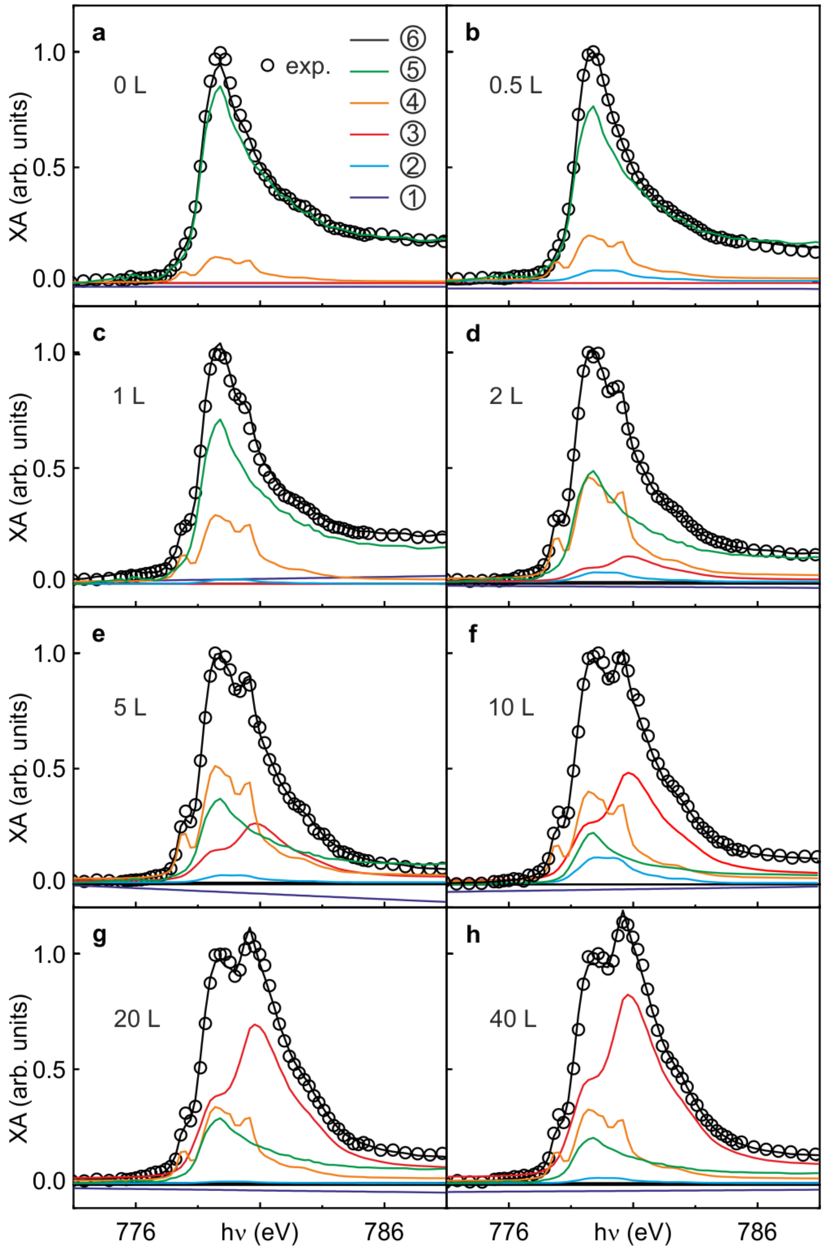

Figure S2: (a-g) Composition of the corresponding XA spectra of Fig. 2(a-g) of the main text as deduced from the fitting procedure described in the Methods section of the main text. (h) XA spectra and composition for 40 L. Experimental XA spectra (circles), (1) linear background term ($A + Bh\nu$), (2) Y(w-CoO), (3) $Y(Co_3O_4)$, (4) Y(CoO), (5) Y(Co), and (6) Y(total).

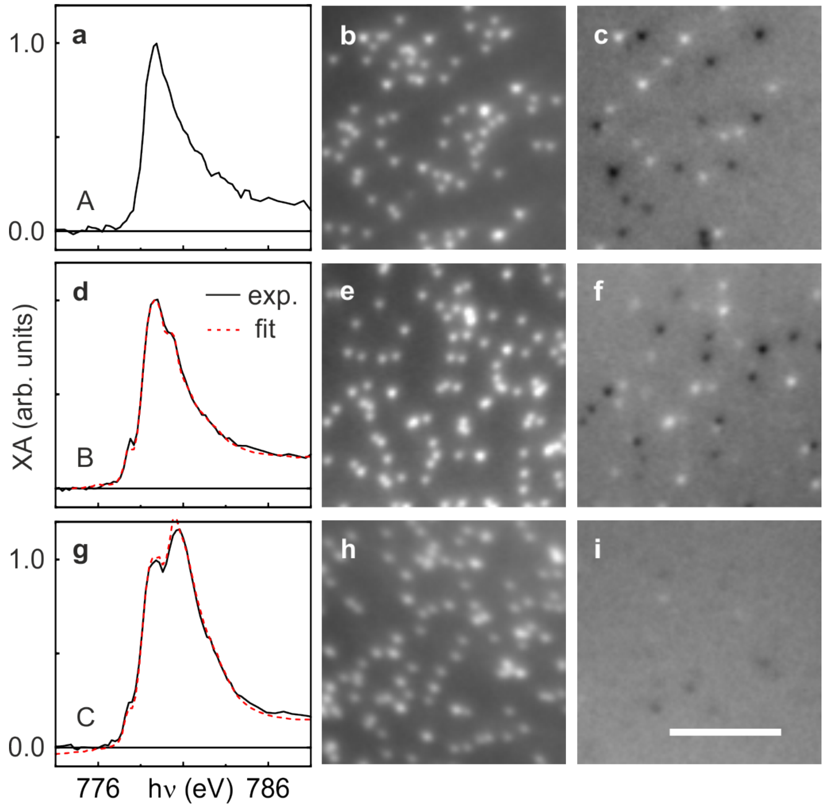

Figure S3: (a) XA spectra of metallic Co nanoparticles (state A) deposited on carbon-capped Si_3N_4-membranes for the HAADF-STEM investigations recorded *in situ* before the final carbon capping. (b) Corresponding elemental contrast and (c) magnetic contrast maps. (d-f) and (g-i) are the corresponding data for Co nanoparticle samples in states B and C. The scale bar is 5 μm. The carbon-capped Si_3N_4-substrates exhibit a lower background intensity in XPEEM and therefore show higher elemental and magnetic contrast as compared to those on Si substrates. As a consequence, in state C a weak magnetic contrast is visible in a larger proportion of nanoparticles when compared to the corresponding data in Fig. 2(o) of the main article for particles deposited on Si. The XA spectral analysis yields a composition of Co ($59 \pm 3\%$), CoO ($31 \pm 3\%$), w-CoO ($0 \pm 3\%$), and Co_3O_4($9 \pm 2\%$) for the sample in state B and Co ($21 \pm 3\%$), CoO ($21 \pm 3\%$), w-CoO ($0 \pm 3\%$), and Co_3O_4($58 \pm 3\%$) for the sample in state C. Fits to the data are shown as red dashed lines. The XMCD contrast in (c,f,e) ranges from ± 0.025.

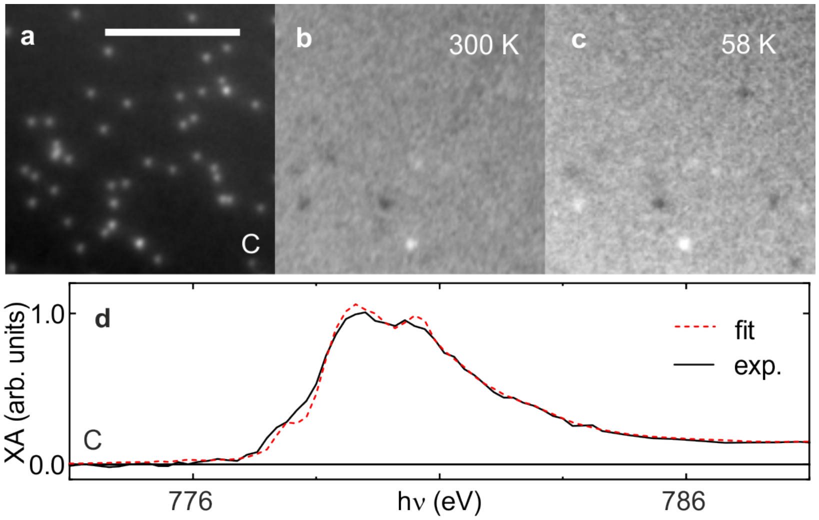

Figure S4: (a) Elemental and (b,c) magnetic XMCD contrast map of Co nanoparticles in state C at 300 K and at 58 K, respectively. The scale bar in (a) is 2 μm and the XMCD contrast in (b,c) ranges from ± 0.020. (d) XA spectra of the nanoparticles (black line) together with a fit to the data (red dashed line). The XA spectral analysis yields a composition of Co ($36 \pm 5\%$), CoO ($35 \pm 4\%$), w-CoO ($0 \pm 5\%$), and Co_3O_4($29 \pm 3\%$).

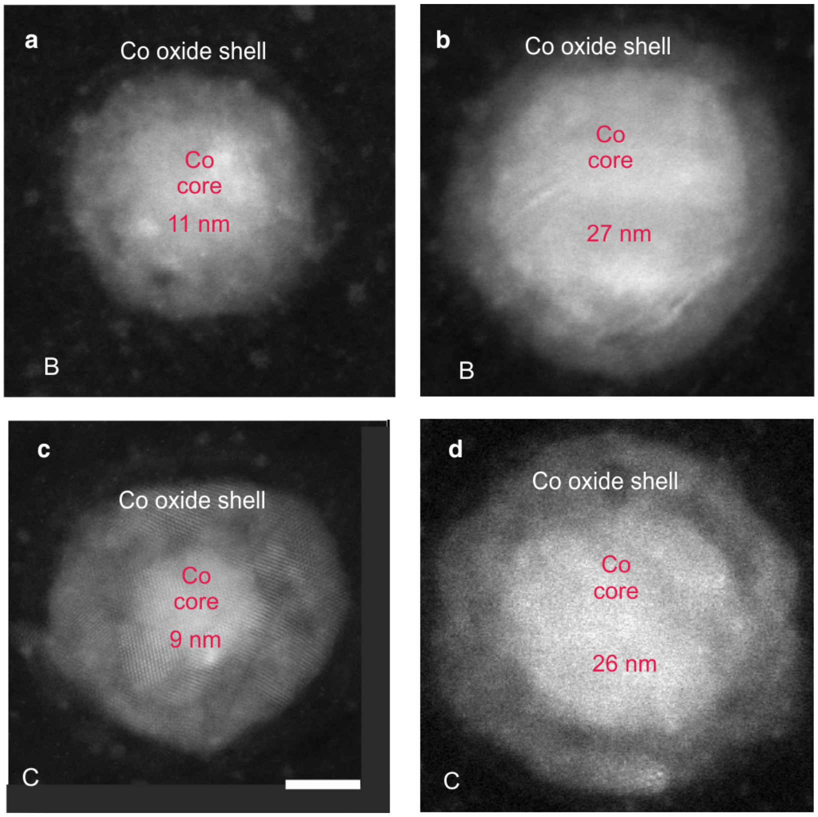

Figure S5: HAADF-STEM images of oxidized nanoparticles with different sizes: (a,b) in state B, (c,d) in state C. The image in (a) is the same as in the uppermost panel in the middle column of Fig. 3 in the main text. The scale bar is 5 nm.

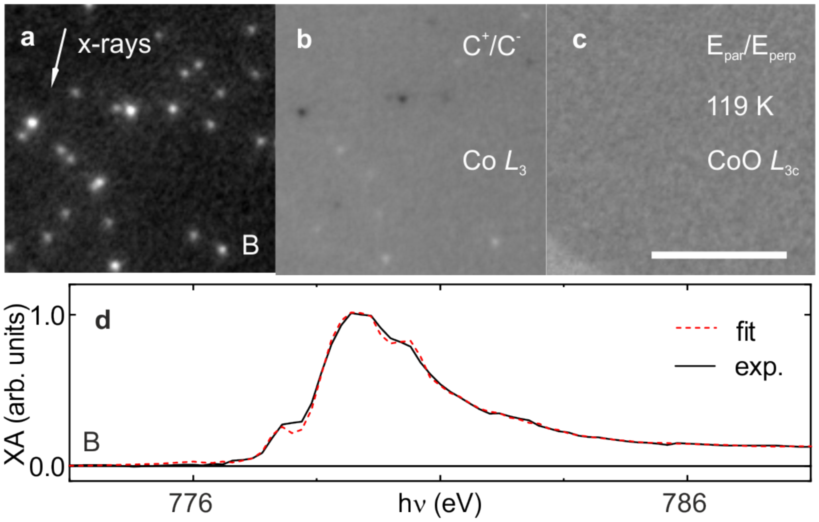

Figure S6: (a) Elemental, (b) magnetic XMCD contrast map of Co nanoparticles in state B as shown in Fig. 5(d) and (h), of the main text, and (c) the corresponding x-ray linear dichroism map at 119 K. The x-ray linear dichroism map is obtained by pixelwise division of two images recorded using x-rays with linear polarization parallel, E_{par}, and perpendicular, E_{perp}, to the substrate surface, with the photon energy set to the CoO L_{3c} feature, see Fig. S1(b). Uniformly antiferromagnetic order across the CoO contribution to the shell would give rise to contrast in the individual nanoparticles ranging from black to white depending on the actual spin lattice orientation due to the XMLD effect.[22] The x-ray propagation direction is indicated by the arrow; the scale bar is $2\,\mu$m and the contrast in (b) and (c) is adjusted to ± 0.025 for comparison. (d) XA spectra of the nanoparticles as shown in Fig. 5(b) in the main text. The latter was recorded with E_{perp} polarization.

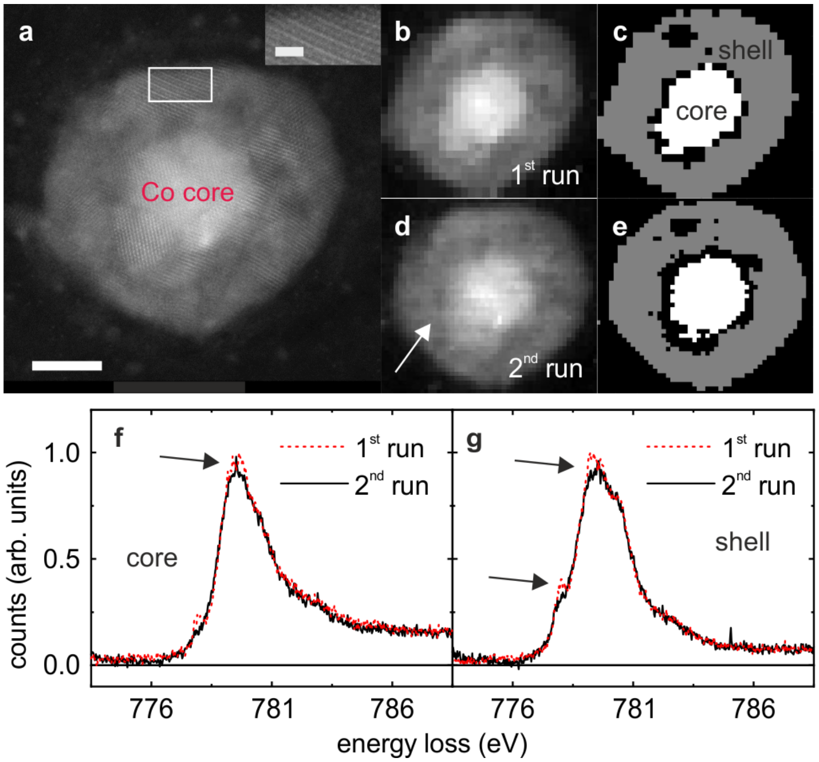

Figure S7: (a) HAADF-STEM image of the oxidized Co nanoparticle shown in Fig. S5(c) in state C with no magnetic contrast at room temperature. The scale bar is 5 nm. The inset is an enlarged view of a Co_3O_4-crystallite (scale bar is 1 nm). (b) First EELS map acquired at an energy loss of 779 eV and (c) regions used for EELS spectra extraction. (d) and (e) similar to (b) and (c), but for a second run. (f) EELS spectra extracted from the core region, showing a dominant metallic Co contribution, and (g) from the shell region, for both runs. When compared to the XA spectra in state C, the EELS spectra exhibit a weaker Co_3O_4 contribution due to the transmission geometry of TEM, which probes the inner and outer parts of the shell of the nanoparticle with similar weights. In addition, we find a sizeable reduction effect originating from the extended electron beam exposure, as seen when comparing the EELS spectra of the first and the second scan, particularly in the regions highlighted by the arrows.

Supplementary References

1. Kleibert, A., Meiwes-Broer, K.-H. & Bansmann, J. Size-dependent magnetic spin and orbital moments of Fe nanoparticles deposited onto Co/W(110). *Phys. Rev. B* **79**, 125423 (2009).

2. Regan, T. J. *et al.* Chemical effects at metal/oxide interfaces studied by x-ray-absorption spectroscopy. *Phys. Rev. B* **64**, 214422 (2001).

3. Bergmann, A. *et al.* Reversible amorphization and the catalytically active state of crystalline Co_3O_4 during oxygen evolution. *Nat. Commun.* **6**, 8625 (2015).

4. Papaefthimiou, V. *et al.* Nontrivial redox behavior of nanosized cobalt: New insights from ambient pressure x-ray photoelectron and absorption spectroscopies. *ACS Nano* **5**, 2182 (2011).

5. Kapoor, A., Yang, R. T. & Wong, C. Surface diffusion. *Catal. Rev.-Sci. Eng.* **31**, 129-214 (1989).

6. Redhead, P. A. Thermal desorption of gases. *Vacuum* **12**, 203-211 (1962).

7. Ono, L. K., Croy, J. R., Heinrich, H. & Cuenya, B. R. Oxygen chemisorption, formation, and thermal stability of pt oxides on Pt nanoparticles supported on SiO_2/Si(001): Size effects. *J. Phys. Chem. C* **115**, 16856–16866 (2011).

8. Barrer, R. M. & Barrie, J. A. Sorption and surface diffusion in porous glass. *Proc. Roy. Soc. A* **213**, 250-265 (1952).

9. Samuel, J., Ottolenghi, M. & Avnir, D. Diffusion-controlled reactions on porous silicas: Mechanisms, surface diffusion coefficients, and effects of geometry. *J. Phys. Chem.* **96**, 6398–6405 (1992).

10. Martin, D. & Duprez, D. Mobility of surface species on oxides. 1. Isotopic exchange of $^{18}O_2$ with ^{16}O of SiO_2, Al_2O_3, ZrO_2, MgO, CeO_2, and CeO_2-Al_2O_3. Activation by noble metals. Correlation with oxide basicity. *J. Phys. Chem.* **100**, 9429–9438 (1996).

11. Sladek, K. J., Gilliland, E. R. & Baddour, R. F. Diffusion on surfaces. II. Correlation of diffusivities of physically and chemically adsorbed species. *Ind. Eng. Chem., Fundam.* **13**, 100–105 (1974).

12. Mutoro, E. *et al.* Electrochemically induced oxygen spillover and diffusion on Pt(111): PEEM imaging and kinetic modelling. *Phys. Chem. Chem. Phys.* **13**, 12798-12807 (2011).

13. Bansal, R. C., Vastola, F. J. & P. L. Walker, J. Studies on ultraclean carbon surfaces II. Kinetics of chemisorption of oxygen on graphon. *Journal of Colloid and Interface Science* **32**, 187-194 (1970).

14. Kelemen, S. R. & Freund, H. O_2 oxidation studies of the edge surface of graphite. *Carbon* **23**, 619-625 (1985).

15. Hock, K. M., Barnard, J. C., Palmer, R. E. & Ishida, H. Competing routes for charge transfer in co-adsorption of K and O_2 on graphite. *Phys. Rev. Lett.* **71**, 641-644 (1993).

16. Incze, A., Pasturel, A. & Chatillon, C. First-principles study of the atomic oxygen adsorption on the (0001) graphite surface and dissolution. *Applied Surface Science* **177**, 226-229 (2001).

17. Sorescu, D. C., Jordan, K. D. & Avouris, P. Theoretical study of oxygen adsorption on graphite and the (8,0) single-walled carbon nanotube. *J. Phys. Chem. B* **105**, 11227–11232 (2001).

18. Bagsican, F. R. *et al.* Adsorption energy of oxygen molecules on graphene and twodimensional tungsten disulfide. *Sci. Rep.* **7**, 1774 (2017).

19. Yang, Z., Yang, N., Yang, J., Bergström, J. & Pileni, M.-P. Control of the oxygen and cobalt atoms diffusion through Co nanoparticles differing by their crystalline structure and size. *Adv. Funct. Mater.* **25**, 891-897 (2015).

20. Bartling, S. *et al.* Pronounced size dependence in structure and morphology of gas-phase produced, partially oxidized cobalt nanoparticles under catalytic reaction conditions. *ACS Nano* **9**, 5984–5998 (2015).

21. Rinaldi-Montes, N. *et al.* Disentangling magnetic core/shell morphologies in Co-based nanoparticles. *J. Mater. Chem. C* **4**, 2302-2311 (2016).

22. Wu, J. *et al.* Direct observation of imprinted antiferromagnetic vortex states in CoO/Fe/Ag(001) discs. *Nat. Phys.* **7**, 303–306 (2011).
