# Supplementary material for: Absence of a pressure gap and atomistic mechanism of the oxidation of pure Co nanoparticles
Source: Nat Commun. 2023 Jan 12;14:174. doi: 10.1038/s41467-023-35846-0 (PMC9837083; doi:10.1038/s41467-023-35846-0)
Supplement: Supplementary file 3 — Description of Additional Supplementary Files [file 41467_2023_35846_MOESM3_ESM.pdf]

**Description of Additional Supplementary Files**

**Supplementary Movie 1**: A movie sequence of Fig. 2(i-o) showing the evolution of the magnetic contrast as a function of oxygen dosage over a larger sample area of 20 μm field of view.
